# Monoacylglycerol Lipase Inhibition Protects From Liver Injury in Mouse Models of Sclerosing Cholangitis

**DOI:** 10.1002/hep.30929

**Published:** 2019-12-30

**Authors:** Matteo Tardelli, Francesca V. Bruschi, Claudia D. Fuchs, Thierry Claudel, Nicole Auer, Victoria Kunczer, Maximilian Baumgartner, Onne A.H.O. Ronda, Henk Jan Verkade, Tatjana Stojakovic, Hubert Scharnagl, Aida Habib, Robert Zimmermann, Sophie Lotersztajn, Michael Trauner

**Affiliations:** ^1^ Hans Popper Laboratory of Molecular Hepatology Division of Gastroenterology and Hepatology Department of Internal Medicine III Medical University of Vienna Vienna Austria; ^2^ Division of Gastroenterology and Hepatology Department of Internal Medicine III Medical University of Vienna Vienna Austria; ^3^ Center for Liver, Digestive and Metabolic Diseases Departments of Pediatrics University Medical Center Groningen University of Groningen Groningen the Netherlands; ^4^ Clinical Institute of Medical and Chemical Laboratory Diagnostics University Hospital Graz Graz Austria; ^5^ Clinical Institute of Medical and Chemical Laboratory Diagnostics Medical University of Graz Graz Austria; ^6^ Université de Paris Centre de Recherche sur l'Inflammation INSERM UMR1149 CNRS ERL 8252 Paris France; ^7^ Department of Biochemistry and Molecular Genetics American University of Beirut Beirut Lebanon; ^8^ Institute of Molecular Biosciences University of Graz Graz Austria

## Abstract

**Background and Aims:**

Monoacylglycerol lipase (MGL) is the last enzymatic step in triglyceride degradation, hydrolyzing monoglycerides into glycerol and fatty acids (FAs) and converting 2‐arachidonoylglycerol into arachidonic acid, thus providing ligands for nuclear receptors as key regulators of hepatic bile acid (BA)/lipid metabolism and inflammation. We aimed to explore the role of MGL in the development of cholestatic liver and bile duct injury in mouse models of sclerosing cholangitis, a disease so far lacking effective pharmacological therapy.

**Approach and Results:**

To this aim we analyzed the effects of 3,5‐diethoxycarbonyl‐1,4‐dihydrocollidine (DDC) feeding to induce sclerosing cholangitis in wild‐type (WT) and knockout (MGL^−/−^) mice and tested pharmacological inhibition with JZL184 in the multidrug resistance protein 2 knockout (*Mdr2^−/−^*) mouse model of sclerosing cholangitis. Cholestatic liver injury and fibrosis were assessed by serum biochemistry, liver histology, gene expression, and western blot characterization of BA and FA synthesis/transport. Moreover, intestinal FAs and fecal microbiome were analyzed. Transfection and silencing were performed in Caco2 cells. MGL^−/−^ mice were protected from DDC‐induced biliary fibrosis and inflammation with reduced serum liver enzymes and increased FA/BA metabolism and β‐oxidation. Notably, pharmacological (JZL184) inhibition of MGL ameliorated cholestatic injury in DDC‐fed WT mice and protected *Mdr2^−/−^* mice from spontaneous liver injury, with improved liver enzymes, inflammation, and biliary fibrosis. *In vitro* experiments confirmed that silencing of MGL decreases prostaglandin E_2_ accumulation in the intestine and up‐regulates peroxisome proliferator–activated receptors alpha and gamma activity, thus reducing inflammation.

**Conclusions:**

Collectively, our study unravels MGL as a metabolic target, demonstrating that MGL inhibition may be considered as potential therapy for sclerosing cholangitis.

**Abbreviations**
AA: arachidonic acid
Abhd6/Abhd12: α/β hydrolase domains 6 and 12
2‐AG: 2‐arachidonoylglycerol
ALT: alanine aminotransferase
Aox: acyl‐coenzyme A oxidase
AP: alkaline phosphatase
AST: aspartate aminotransferase
ATP: adenosine triphosphate
BA: bile acid
BEC: biliary epithelial cell
Bsep: bile salt export pump
CD: cluster of differentiation
CDCA: chenodeoxycholic acid
Ck19: cytokeratin 19
Col1α1/Col1α2: collagen types 1α 1 and 2
Cox2: cyclooxygenase‐2
Cpt1α: carnitine palmitoyltransferase 1A
Cyp7a1: cytochrome P450 7A1
DDC: 3,5‐diethoxycarbonyl‐1,4‐dihydrocollidine
DMEM: Dulbecco's modified Eagle's medium
FA: fatty acid
FBS: fetal bovine serum
Fgf: fibroblast growth factor
FXR: farnesoid X receptor
FXRE: FXR response element
h: human
H&E: hematoxylin and eosin
Hmox1/HO‐1: heme oxygenase 1
IHC: immunohistochemistry
IHH: immortalized human hepatocyte
LPS: lipopolysaccharide
Mac‐2: galectin‐3
Mcp1: monocyte chemoattractant protein 1
Mdr2: multidrug resistance protein 2
MGL: monoacylglycerol lipase
Mrp2/3/4: adenosine triphosphate binding cassette subfamily C member 2/3/4
NR: nuclear receptor
Nrf2: nuclear factor erythroid 2–related factor 2
Ntcp: sodium‐taurocholate cotransporting polypeptide
Oatp1: ornithine aminotransferase pseudogene 1
Opn: osteopontin
PBC: primary biliary cholangitis
Pgc1α: peroxisome proliferator–activated receptor‐gamma coactivator 1 alpha
PGE_2_: prostaglandin E2
PG‐G: prostaglandin glycerol ester
Ppar: peroxisome proliferator–activated receptor
PSC: primary sclerosing cholangitis
RXR: retinoid X receptor
si: small interfering
Srebp1c/Srebp2: sterol regulatory element‐binding proteins 1c and 2
TG: triglycerides
Tgfβ: transforming growth factor beta
Tnfα: tumor necrosis factor alpha
Vcam‐1: vascular cell adhesion protein 1
WT: wild type

Cholangiopathies such as primary sclerosing cholangitis (PSC) and primary biliary cholangitis (PBC) are chronic hepatobiliary disorders characterized by accumulation of bile acids (BAs) in the liver, leading to hepatocellular necrosis, progressive fibrosis, and end‐stage liver disease.^1^, ^2^ Current therapeutic approaches for treating cholestasis mainly rely on the use of ursodeoxycholic acid (UDCA) as first‐line treatment; however, UDCA has no proven efficacy for PSC, and only a proportion of patients with PBC show a sufficient response.^3^ Monoacylglycerol lipase (MGL) is the rate‐limiting enzyme in the degradation of monoacylglycerols.^4^ MGL hydrolyzes monoacylglycerols deriving from phospholipids or triglycerides (TG) into glycerol and fatty acids (FAs),^4^, ^5^ with highest expression in the brain, white adipose tissue, and liver.^6^ In addition, to its role in lipid metabolism, MGL is a pivotal component of the endocannabinoid system as it hydrolyzes 2‐arachidonoylglycerol (2‐AG), an endogenous ligand for the cannabinoid receptors, into arachidonic acid (AA).^7^ Studies in MGL knockout (MGL^−/−^) mice suggested a key role for MGL in metabolic processes and energy homeostasis.^8^ We previously reported that MGL^−/−^ mice fed a high‐fat diet gained less body weight compared to wild‐type (WT) animals.^9^ Other studies showed that in transgenic mouse models MGL overexpression in the forebrain resulted in a leaner phenotype,^10^ whereas overexpression in the intestine caused fat accumulation and increased food intake.^11^ Collectively, these reports depict contradictory roles of MGL in the brain and peripheral tissues. Recently, our work investigated the effect of MGL deficiency on liver fibrosis development.^12^ Interestingly, lack of MGL promoted fibrosis regression due to autophagy‐mediated anti‐inflammatory properties in macrophages.^12^ Furthermore, Cao et al. showed that global genetic and pharmacological inhibition of MGL protects against inflammation and liver lesions induced by ischemia/reperfusion injury.^13^ However, the specific role of MGL and its metabolites as potential drivers of cholestatic liver diseases such as PBC and PSC is unknown. In the present study, we aimed to explore the role of MGL in the development of cholangitis and associated complications. By feeding MGL^−/−^ mice a diet containing 0.1% of 3,5‐diethoxycarbonyl‐1,4‐dihydrocollidine (DDC) and treating WT and multidrug resistance protein 2 knockout (*Mdr2^−/−^*) mice with MGL inhibitor (JZL184), we analyzed the effects of absent MGL activity using these two murine models of PSC. Our results indicate that MGL is involved in the development of cholestasis and cholangitis as its genetic deletion in mice protected from biliary fibrosis and inflammation induced by DDC feeding. Notably, pharmacological (JZL184) inhibition of MGL also ameliorated DDC‐induced cholestasis and protected *Mdr2^−/−^* mice from spontaneous liver injury improving liver enzymes, inflammation, and biliary fibrosis. Further, we report a possible role for MGL and its metabolites in the gut–liver axis, where modulation of the nuclear receptors (NRs) peroxisome proliferator–activated receptor alpha (PPAR‐α) and PPAR‐γ activity by AA diminishes intestinal inflammation and prostaglandin E_2_ (PGE_2_) content. Overall, our results uncover a potential role of MGL inhibition in the preservation of liver function and the gut–liver axis in cholestatic diseases such as PSC, a disease so far lacking effective pharmacological therapy.

## Materials and Methods

### Animal Experiments

Experiments were performed in 3‐month‐old male MGL^−/−^ mice and WT littermates (C57BL/6J background, n = 8 per group unless stated otherwise) weighing 25‐30 g, generated by R. Zimmermann,^14^ University of Graz. Mice included in all the experimental setting were littermates bred from a heterozygous colony. All mice were housed in a 12‐hour light/dark house facility with *ad libitum* consumption of water and food. They received either a DDC diet (A04 chow diet supplemented with 0.1% DDC) or standard chow (A04; both from SAFE Diets, Augy, France) for 2 weeks. *Mdr2^−/−^* mice (FVBN background, n = 9 per group; Jackson Laboratory, Bar Harbor, ME) were housed in a 12‐hour light/dark house facility with water and standard chow diet (A04) *ad libitum*. Eight‐week‐old *Mdr2^−/−^* mice received either a control diet (A04) or a diet supplemented with 2% of JZL184 (A04 + JZL184) for 4 weeks. WT mice received 2 weeks of DDC feeding and 2% of JZL184 or chow for 4 days to investigate disease resolution. The experimental protocols were approved by the local Animal Care and Use Committee (BMWF.66.009/0117‐II/3b/2013), and routine serum biochemical analysis was performed as described.^15^

### Liver Histology, Immunohistochemistry, and Immunofluorescence

Livers were fixed in 4% neutral buffered formaldehyde solution for 24 hours and embedded in paraffin. Sections of 3 μm thickness were stained with hematoxylin and eosin (H&E) or sirius red as described.^16^ For immunohistochemistry (IHC), slides were blocked for 60 minutes in blocking buffer (1 × phosphate‐buffered saline [PBS], 5% goat serum and 0.3% Triton X‐100).^17^ Blocking buffer was aspirated, and sections were incubated overnight at 4°C with an antimouse osteopontin (OPN) antibody (R&D Systems) for OPN detection, vascular cell adhesion protein 1 (Vcam‐1), and Mac‐2 (Santa Cruz Biotechnology). Afterward, slides were washed 3 times in 1 × PBS and incubated for 1 hour at room temperature with the respective horseradish peroxidase–labeled secondary antibody (Dako). Slides were then washed and developed with 3‐amino‐9‐ethylcarbazole‐based or 3,3′‐diaminobenzidine‐based solution and counterstained with hematoxylin. For immunofluorescence, slides were incubated with a cluster of differentiation 11b (CD11b) peridinin chlorophyll protein, cyanine 5.5–labeled antimouse (Mac‐1α chain, M1/70) (BD Biosciences) and counterstained with 4′,6‐diamidino‐2‐phenylindole (Sigma). Relative quantification of immunofluorescent staining was performed using Image J software in an automated fashion.^18^

### Cell Culture

Caco‐2 cells were cultured in Dulbecco's modified Eagle's medium (DMEM) with 4.5 g/L glucose supplemented with 20% fetal bovine serum (FBS), L‐glutamine (0.2 mol/L), and nonessential amino‐acids and antibiotics (all Thermo Fisher Scientific). Immortalized human hepatocytes (IHHs) were cultured in DMEM + 10% FBS, LX2 cells in DMEM + 5% FBS, small biliary epithelial cells (BECs) in DMEM + 10% FBS, and U937 cells in Roswell Park Memorial Institute medium + 10% FBS and vitamin D (2.5 ng/mL). After confluence, cells were silenced for MGL with Lipofectamine 2000 Transfection Reagent (Thermo Fisher Scientific) and small interfering (si) MGL RNA at a concentration of 5 µM (Santa Cruz Biotechnology) according to the manufacturer's instructions. The efficiency of transfection was evaluated through quantitative RT‐PCR and western blot. Thereafter, cells were treated with either 100 µM AA or 75 µM chenodeoxycholic acid (CDCA) for 6 hours without serum.

### Luciferase Assay

Caco2 cells, IHHs, LX2 cells, BECs, and U937 cells were seeded in a 24‐well plate and transiently transfected with 0.3 µg/well of the plasmids encoding human farnesoid X receptor (FXR). Human (h) PPAR‐α, hPPAR‐γ, and hRXRα (provided by Philippe Lefebvre, Institut Pasteur de Lille, Lille, France) and the FXR or PPAR‐luciferase (provided by Peter Young Dupont, Oakley, CA) using Fugene transfection reagent (Promega, Madison, WI) were cultured in sterile OptiMeM for 12 hours. Complete medium was replaced for an additional 24 hours, and cells were lysed using a lysis solution (4% Triton X‐100, glycyl‐glycine 100 mM, MgSO_4_ 100 mM, ethylene glycol tetraacetic acid 250 mM) for 1 hour at room temperature on a rocking platform. The extracts were then combined with solution containing the substrate (luciferin 2.5 mM and adenosine triphosphate [ATP] 20 mM; Sigma‐Aldrich), and luciferase activity was measured with a luminometer (Lumat LB9507; EG&G Berthold, Germany).

### Western Blot Analysis

Tissues were collected in radio immunoprecipitation assay buffer, and the protein concentration was measured using a 660‐nm protein assay kit. Protein extracts were processed for crude membrane isolation and loaded on sodium dodecyl sulfate–polyacrylamide gel electrophoresis using 10% polyacrylamide gels to investigate expression of the transporters heme oxygenase 1 protein (HO‐1), ornithine aminotransferase pseudogene 1 (Oatp1), sodium‐taurocholate cotransporting polypeptide (Ntcp), ATP binding cassette subfamily C member 2/3/4 (Mrp2/3/4), bile salt export pump (Bsep), MGL (all 1:500; Santa Cruz Biotechnology), and calnexin (1:1,000).

### Gas Chromatography

Liver and intestine samples (approximately 10 mg) were subjected to FA isolation and derivatization. All TGs, phospholipids, and cholesterol esters were split up into free FAs and derivatized by the methyl donor (acidic methanol). A known quantity of C17 was added to each sample as internal standard. From the internal standard, we calculated the values of each FA species. After methylation, the sample was concentrated in hexane and injected into the gas chromatography system.

### 16S Ribosomal RNA Microbiome Analysis

Amplicon sequencing of the 16S V3‐V4 region was performed using MiSeq technology and standard Illumina protocols. Sequencing reads were processed with deficiency of adenosine deaminase 2 and seven in absentia.^19^ Statistical analysis was done using R; to analyze the similarity of microbial profiles, UniFrac and the vegan package adonis were used.^20^ The Kruskal‐Wallis and Wilcoxon (for pairwise comparison) rank sum tests were used to compare bacterial abundances.

### Statistical Analysis

All data are expressed as mean ± SD. Statistical analyses were performed using the Mann‐Whitney test with Prism software (GraphPad, CA), unless otherwise stated. *P* ≤ 0.05 was considered statistically significant.

For additional methods see Supporting Information.

## Results

### MGL Deletion Attenuates Cholestatic Liver and Bile Duct Injury Induced by DDC Feeding

**Figure 1:**
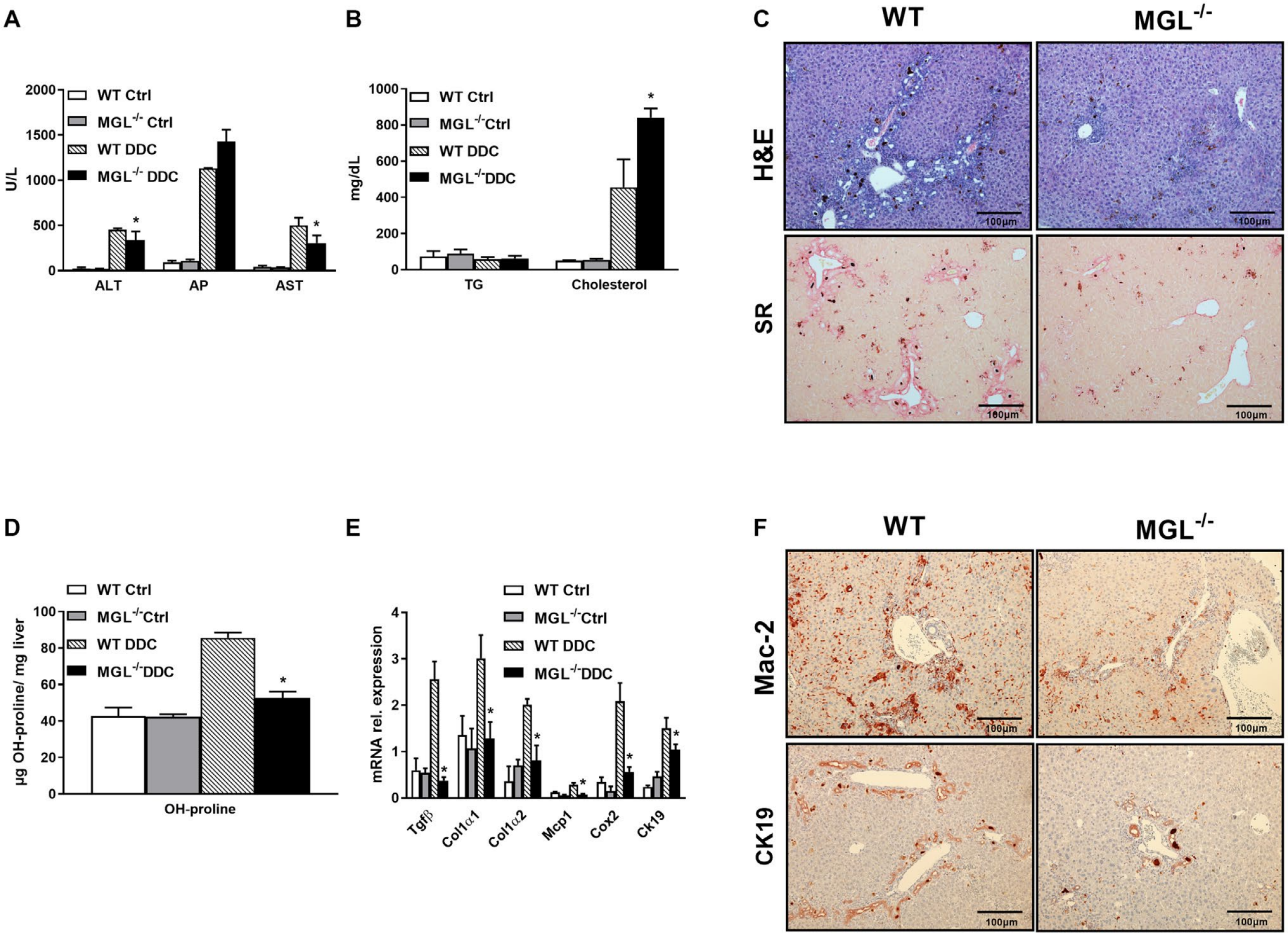
MGL^−/−^ mice display reduced biliary fibrosis and inflammation after 2 weeks of DDC feeding. Cholestatic liver injury resembling PSC was induced in C57BL/6J and MGL^−/−^ mice (n = 8 per group) by 2 weeks feeding with DDC. (A) Serum biochemistry reflects improved levels of transaminases (ALT and AST but unchanged AP) as well as (B) increased cholesterol levels and unchanged TG. (C) Representative H&E images (×10 magnification) with markedly improved liver histology and ameliorated fibrosis in line with (D) reduced hepatic hydroxy (OH)‐proline levels in MGL^−/−^ mice fed DDC. (E) Hepatic gene expression of profibrogenic markers *Tgfβ*, *Col1α1*, and *Col1α2* diminished while proinflammatory cytokines/chemokines *Mcp1*, *Cox2*, and *Ck19* were reduced after DDC feeding. (F) Representative images of Mac‐2 and CK19 staining in liver tissue. White/gray bars represent mice fed the control diet; black and dashed bars represent mice fed the DDC diet. Results are expressed as mean ± SD. *P < 0.05 for MGL^−/−^ DDC versus WT DDC mice. Abbreviations: Ctrl, control; SR, sirius red.

We used the DDC diet as an established model to induce sclerosis cholangitis in WT and MGL^−/−^ mice. Notably, MGL genetic ablation was protective against DDC‐induced sclerosis cholangitis, as shown by diminished serum levels of alanine aminotransferase (ALT) and aspartate aminotransferase (AST), without changes in alkaline phosphatase (AP) (Fig. 1A) but (trend‐wise) increased serum BAs (Supporting Fig. S1A). DDC also increased total cholesterol but not TG serum levels in MGL^−/−^ mice (Fig. 1B). Furthermore, MGL deletion conserved liver architecture in H&E staining (Fig. 1C), diminishing ductular reaction and preventing the onion skin–type lesions characteristic of sclerosing cholangitis seen in WT animals challenged with DDC (Fig. 1C, H&E, WT). Biliary fibrosis was also attenuated as shown by sirius red staining (Fig. 1C) and by quantification of hepatic hydroxyproline (OH‐proline) content (Fig. 1D). In line with this, gene expression analysis revealed a reduction of fibrotic markers such as tumor growth factor beta (*Tgfβ*) and collagen type 1 α1 and α2 (*Col1α1*, *Col1α2*) (Fig. 1E). MGL deletion also improved hepatic Inflammation as reflected by the decreased amount of *F4/80* gene expression (Supporting Fig. S1B) and Mac‐2 and CD11b IHC staining (Fig. 1F; quantification of CD11b Supporting Fig. S1C). Proinflammatory markers such as monocyte chemoattractant protein 1 (*Mcp1*) and cyclooxygenase‐2 (*Cox2*) were also diminished in the liver of DDC‐treated MGL^−/−^ compared to WT mice (Fig. 1E). Importantly, OPN, Vcam‐1, and cytokeratin 19 (Ck19), which are markers for proliferative bile ducts and reactive cholangiocyte phenotypes, were decreased in MGL^−/−^ mice after DDC feeding (Fig. 1E,F; Supporting Fig. S1B‐D). Altogether, our data highlight that MGL deletion protected liver and bile ducts from DDC‐induced damage.

### Lack of MGL Increases FA Synthesis/β‐Oxidation and Affects BA Metabolism/Transport

**Figure 2:**
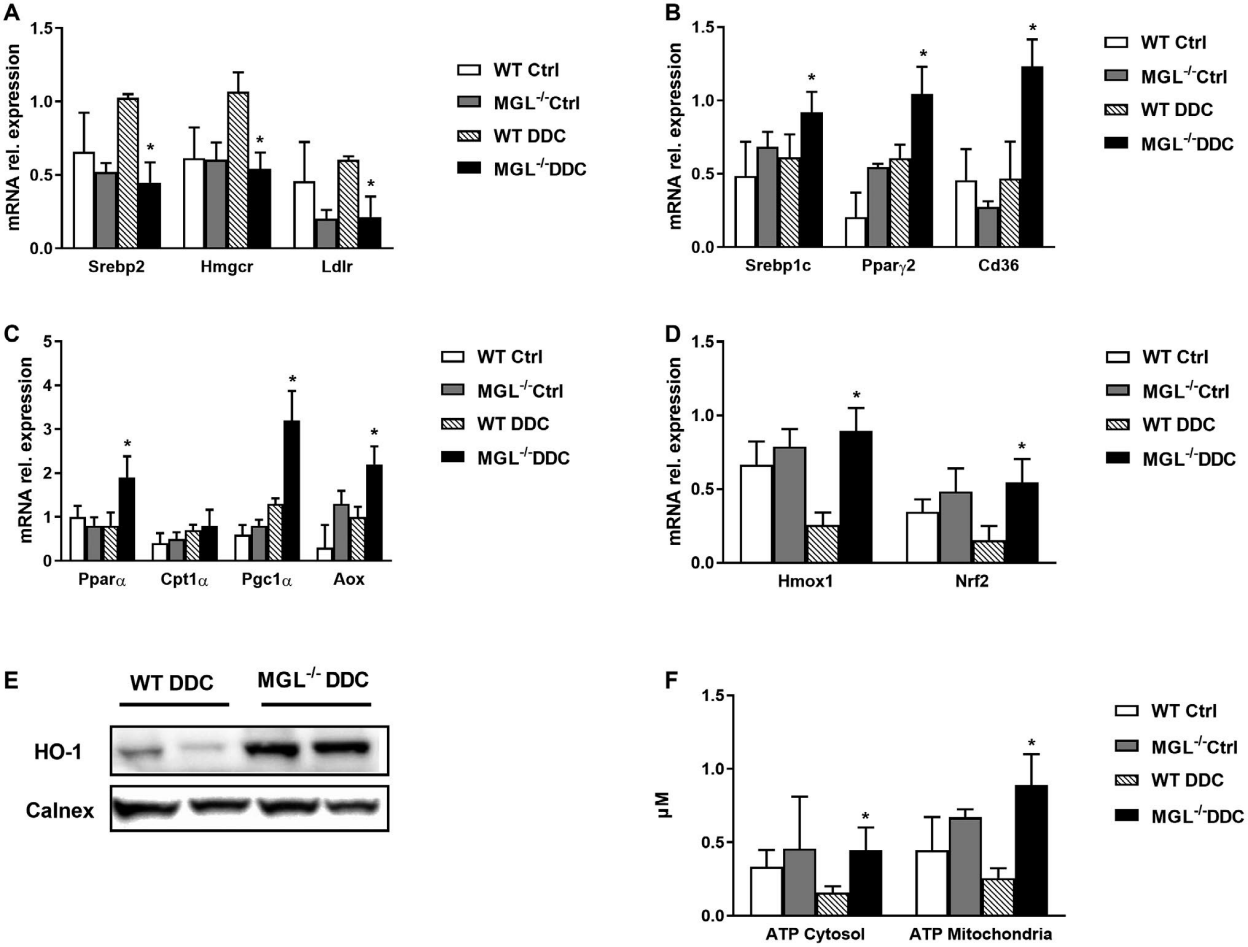
MGL deletion down‐regulates cholesterol synthesis while increasing FA synthesis and oxidation. (A) Hepatic gene expression of *Srebp2*, *Hmgcr*, and *Ldlr* indicates diminished cholesterol synthesis. (B) Hepatic expression of FA synthesis genes increased as seen for *Srebp1c*, *Pparγ2*, and *Cd36* gene. (C) Mitochondrial β‐oxidation increased as evidenced by *Pparα*, *Cpt1α*, *Pgc1α*, *Aox*, and (D) *Hmox1* and *Nrf2* gene expression. In line with (E) western blot analysis of HO‐1 (calnexin shown as loading control). (F) Hepatic ATP content measured in cytosol and mitochondria from liver homogenates increased in MGL^−/−^ DDC. Results are expressed as mean ± SD. *P < 0.05 for MGL^−/−^ DDC versus WT DDC mice (n = 8). Abbreviation: Ctrl, control.

Next, we investigated the impact of MGL deletion on lipid metabolism under baseline and DDC challenge. Because MGL‐deficient mice had higher serum levels of cholesterol despite normal TG (Fig. 1B), we analyzed key players in lipid and cholesterol metabolism. Notably, cholesterol synthesis was decreased as shown by sterol regulatory element‐binding protein 2 (*Srebp2*), 3‐hydroxy‐3‐methyl‐glutaryl‐coenzyme A reductase (*Hmgcr*), and low‐density lipoprotein receptor (*Ldlr*) gene expression (Fig. 2A). Moreover, FA synthesis and uptake increased as reflected by *Srepb1c*, *Pparγ2*, and its target gene *Cd36* (Fig. 2B), highlighting an adaptive response to FA/cholesterol species within the liver. *Pparδ* expression remained unchanged, but its target gene, Lipin 2, was significantly up‐regulated (Supporting Fig. S1E). Because others have reported mitochondrial dysfunctions already after 2 weeks of DDC feeding,^21^ we analyzed key mitochondrial genes involved in FA oxidation. *Pparα* and its target genes peroxisome proliferator–activated receptor‐gamma coactivator 1 alpha (*Pgc1α*) and acyl‐coenzyme A oxidase (*Aox*) were up‐regulated, while carnitine palmitoyltransferase 1A (*Cpt1α*) remained unchanged (Fig. 2C). Interestingly, heme oxygenase 1 (*Hmox1)* gene and protein (HO‐1) expression up‐regulated together with the nuclear factor erythroid 2–related factor 2 (*Nrf2*) gene in MGL^−/−^ mice (Fig. 2D,E), indicating an antioxidant response to DDC intoxication. Moreover, hepatic ATP content was increased in cytosol and mitochondria of MGL^−/−^ mice fed DDC, further demonstrating a response to compromised oxidative phosphorylation by mitochondria (Fig. 2F).

**Figure 3:**
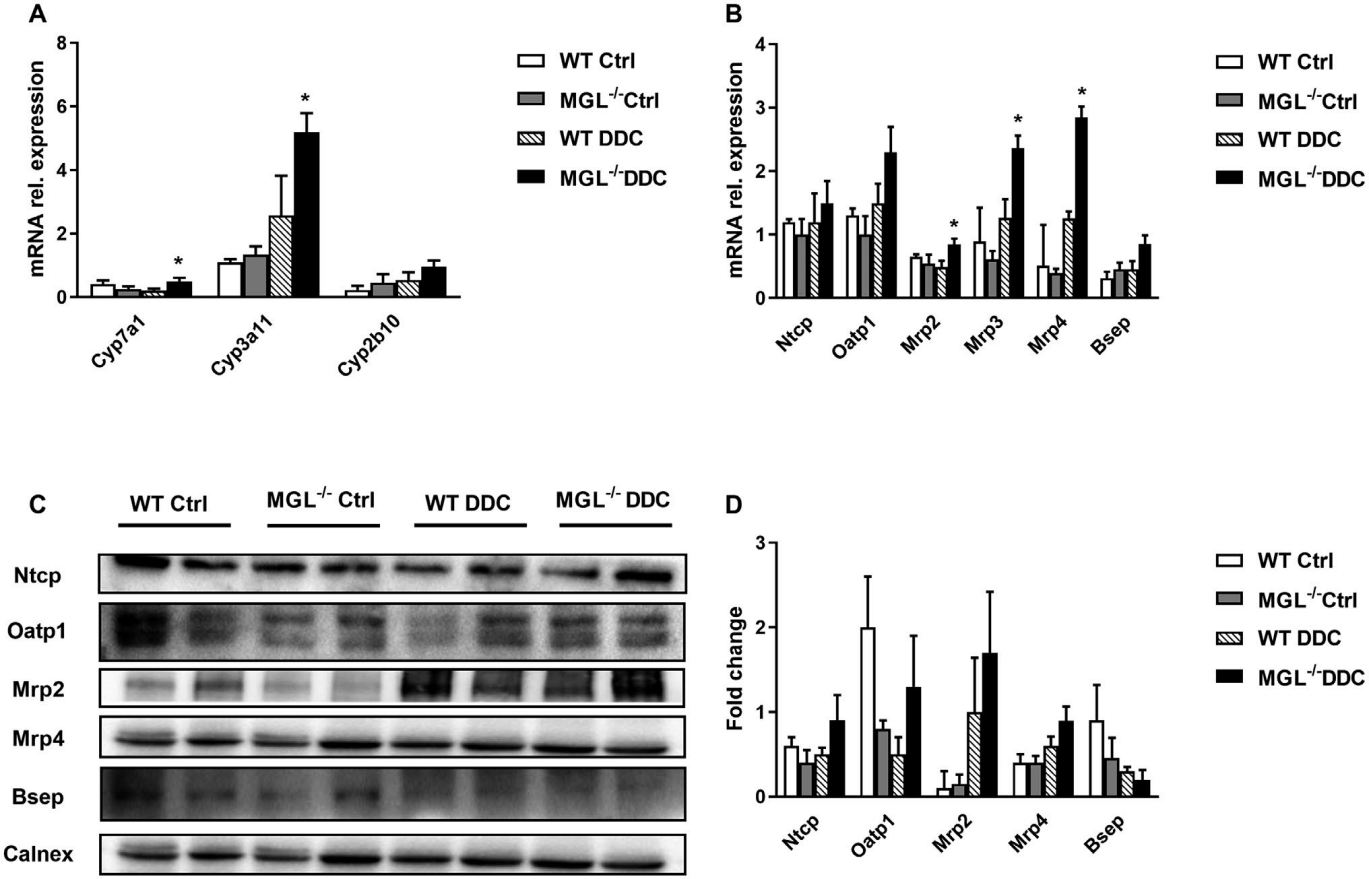
BA synthesis and export are increased, whereas import is unchanged in MGL^−/−^ mice fed DDC. (A) Hepatic gene expression of limiting BA synthesis pathway (*Cyp7a1*) and detoxification (*Cyp3a11, Cyp2b10*). (B) Gene expression profiling of *Ntcp, Oatp1, Mrp2, Mrp3, Mrp4, Bsep.* (C) Representative western blot with (D) corresponding densitometry (n = 2 per group) of BA transporters showing augmented Mrp2, unchanged secretion (Bsep), and unchanged uptake (Oatp1 and Ntcp), Calnexin was used as a loading control. Results are expressed as mean ± SD. *P < 0.05 for MGL^−/−^ DDC versus WT DDC mice (n = 8). Abbreviation: Ctrl, control.

We next addressed whether MGL deletion had an impact on BA homeostasis. Notably, cytochrome P450 7A1 (*Cyp7a1*) expression was increased (Fig. 3A), and BA detoxification was also up‐regulated for *Cyp3a11* and in trend for *Cyp2b10* (Fig. 3A) in MGL^−/−^ mice fed DDC versus WT DDC. In line, DDC treatment increased the plasma level of BAs to a higher extent in MGL^−/−^ versus WT mice (Supporting Fig. S1A) and induced the gene expression of canalicular *Mrp2* in MGL^−/−^ mice (Fig. 3B). Moreover, the basolateral transporters *Mrp3* and *Mrp4* were increased in DDC‐fed MGL^−/−^ versus WT mice, while *Bsep* and basolateral *Ntcp/Oatp1* remained unchanged (Fig. 3B) together with ileal apical sodium‐dependent bile acid transporter and organic solute transporter alpha/beta (Supporting Fig. S1F). Western blot analysis showed increased uptake transporters Ntcp/Oatp1, increased Mrp2/Mrp4, and decreased Bsep (Fig. 3C,D). Collectively, MGL deletion down‐regulated cholesterol synthesis and increased FA uptake/oxidation and BA transport while ameliorating mitochondrial dysfunction induced by DDC feeding.

### Intestinal Inflammation Diminishes in MGL^−/−^ Mice Despite AA Accumulation

**Figure 4:**
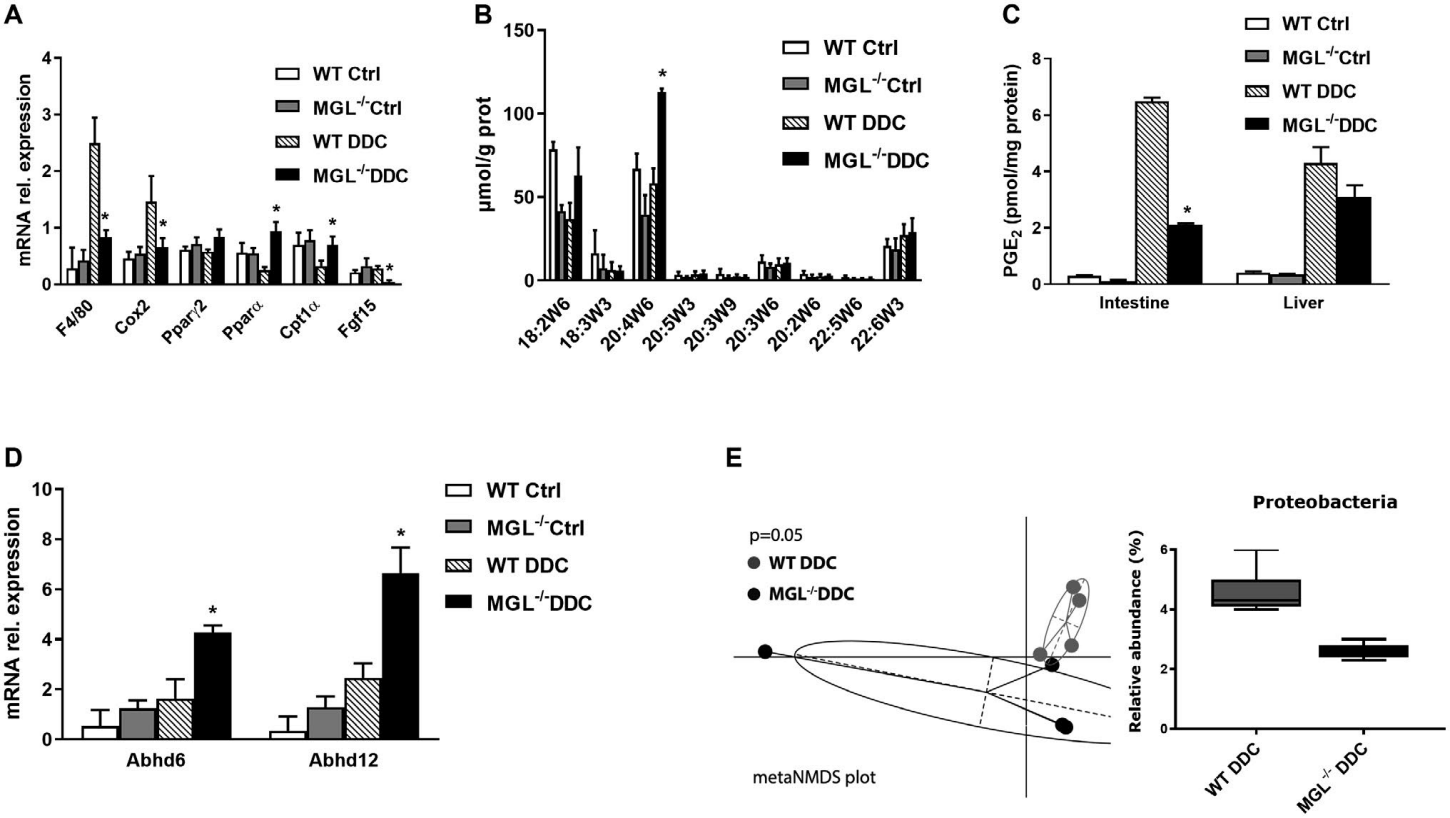
Intestinal inflammation diminishes in MGL^−/−^ mice despite linoleic acid and AA accumulation. (A) Intestinal gene expression of proinflammatory genes *Tnfα*, *F4/80*, and *Cox2* decreased in MGL^−/−^ mice, whereas *Pparγ*, *Pparα*, and *Cpt1α* increased despite *Fgf15* down‐regulation. (B) Gas chromatography quantification of intestinal polyunsaturated FAs evidenced accumulation of 18:2w6 (linoleic acid) and 20:4w6 (AA) in MGL^−/−^ mice fed DDC (n = 4). (C) PGE_2_ levels decreased in intestine and liver (trend wise). (D) Intestinal gene expression of alternative lipid‐hydrolyzing enzymes *Abhd6* and *Abhd12* were up‐regulated in MGL^−/−^ mice fed DDC. (E) DDC treated MGL^−/−^ mice showed significantly different microbiome from WT DDC (dot plot) with reduced abundance of Proteobacteria (bar graph). Results are expressed as mean ± SD. *P < 0.05 for MGL^−/−^ DDC versus WT DDC mice (n = 8). Abbreviation: Ctrl, control.

We further aimed to understand whether the protective effects in liver seen with MGL inactivation also affected intestinal homeostasis. Intestinal inflammatory markers tumor necrosis factor alpha (*Tnfα*), *F4/80*, and *Cox2* were profoundly decreased in MGL^−/−^ mice (Fig. 4A) at the gene expression level as well as at the protein level in IHC for Mac‐2 (Supporting Fig. S2A). While gene expression of *Pparα*, *Cpt1α*, and *Pparγ2* (in trend) increased, fibroblast growth factor 15 (*Fgf15*) was diminished (Fig. 4A), in line with diminished local inflammation and *Cyp7a1* induction/increased BA synthesis in the liver. In order to understand whether different lipid mediators deriving from MGL deletion had a role in intestinal inflammation, we measured the FA content in liver and intestine as pivotal ligands for NRs. Notably, linoleic acid (18:2w6, in trend) and AA (20:4W6) accumulated in the intestine of MGL^−/−^ mice fed the DDC diet (Fig. 4B), when no other polysaturated or monosaturated FAs accumulated in the intestine (not shown). In the liver no significant differences were found apart from decreased palmitic acid (16:0) in DDC‐fed MGL^−/−^ mice (not shown). Because AA is converted to the proinflammatory eicosanoid PGE_2_ through COX2, we measured PGE_2_ content, uncovering a decrease in the intestine, liver (in trend) (Fig. 4C), and plasma (Supporting Fig. S2B) of MGL^−/−^ mice fed DDC versus WT DDC. Because other hydrolases are known to convert 2‐AG to AA, we measured their gene expression, noting an up‐regulation of α/β hydrolase domains 6 and 12 (*Abhd6* and *Abhd12*), probably accounting for the increased amount of AA despite MGL invalidation (Fig. 4D). To investigate the effect of MGL deletion on the intestinal microbiome, 16S amplicon sequencing of fecal DNA samples was performed. DDC‐treated MGL^−/−^ mice showed significantly different microbiome in comparison to DDC‐treated WT mice, with reduced abundance of Proteobacteria (Fig. 4E).

### Pharmacological Blockade of MGL (JZL184) IN *Mdr2^−/−^* Ameliorates Biliary Fibrosis and Inflammation

**Figure 5:**
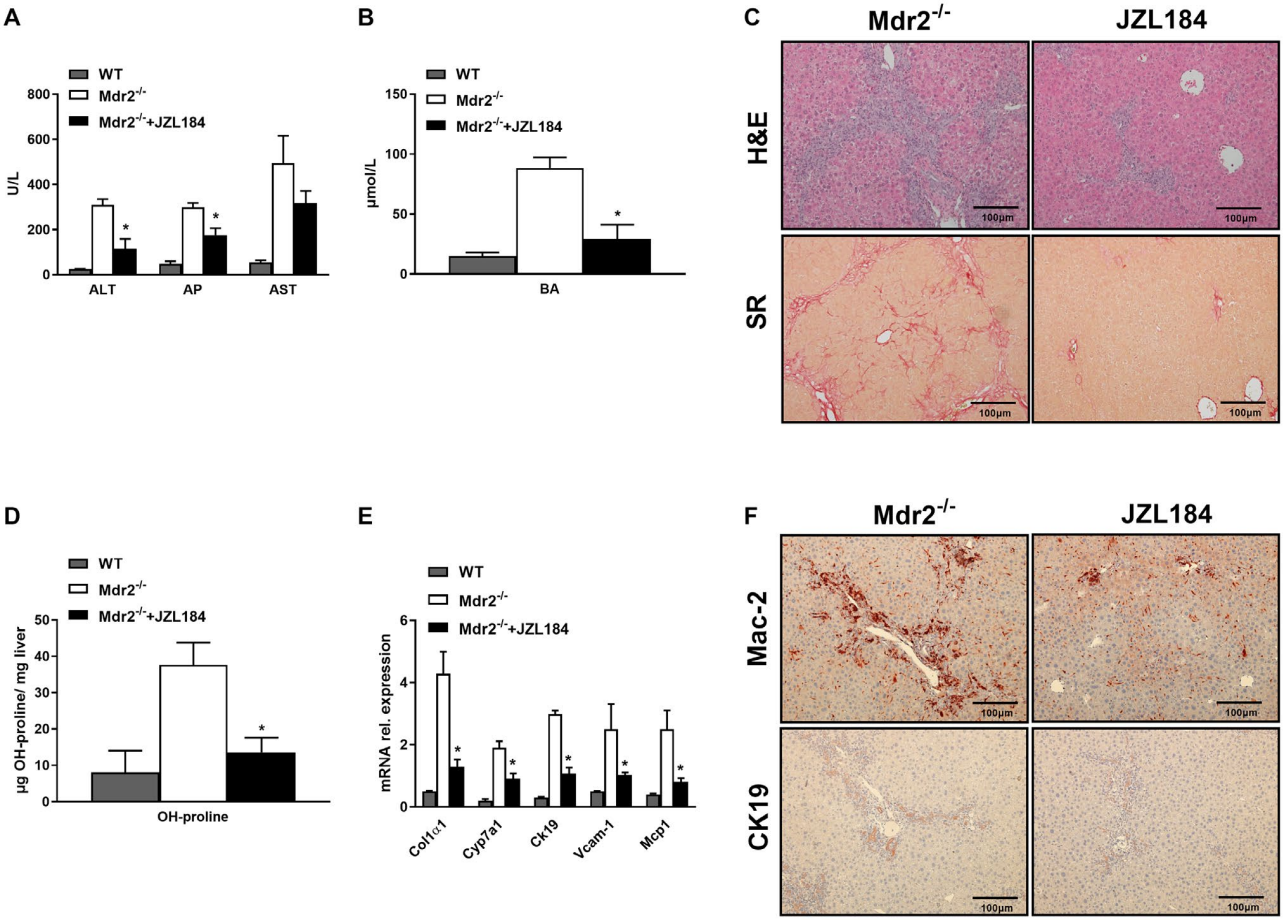
The MGL inhibitor JZL184 ameliorates biliary fibrosis and inflammation in *Mdr2^−/−^* mice. Eight‐week‐old *Mdr2^−/−^* mice were fed JZL184 for 4 weeks (n = 9 per group). (A) Serum biochemistry evidenced decreased serum levels of ALT and AP, with unchanged AST and (B) diminished plasma BA in JZL184‐fed mice but not in *Mdr2^−/−^* mice receiving chow. (C) Representative H&E images (×10 magnification) with markedly improved liver histology and ameliorated fibrosis (sirius red) confirmed by (D) diminished hepatic OH‐proline levels. (E) Hepatic gene expression of *Col1α1*, *Cyp7a1*, *Ck19*, and *Vcam‐1* and proinflammatory cytokine *Mcp1* diminished*.* (F) Representative Mac‐2 and CK19 IHC pictures show reduced cholangiocyte proliferation and inflammation in JZL184‐treated *Mdr2^−/−^* mice. Results are expressed as mean ± SD. *P < 0.05 for *Mdr2^−/−^* in white bars versus *Mdr2^−/−^* mice fed JZL184 in black bars and WT gray bars. Abbreviation: SR, sirius red.

**Figure 6:**
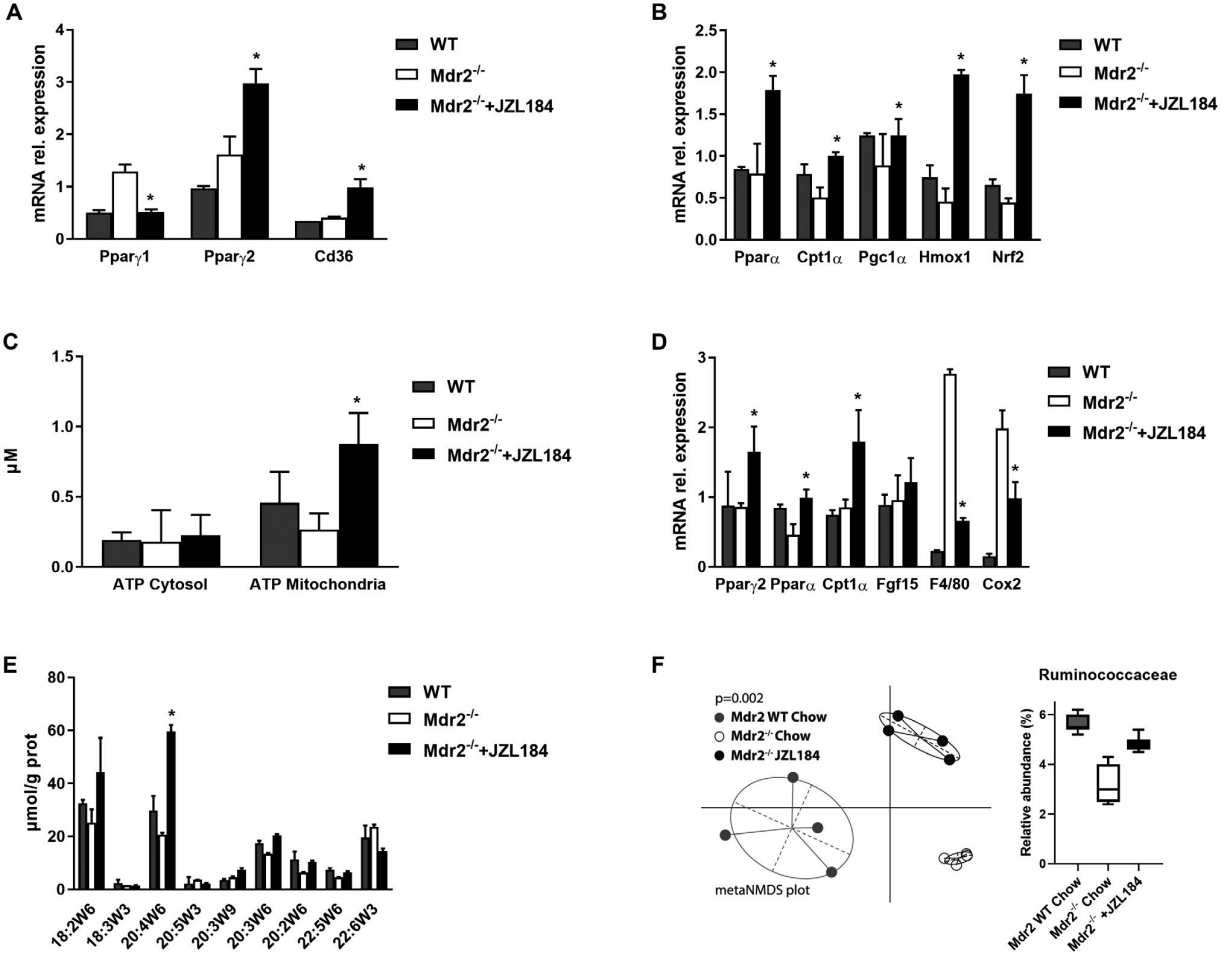
JZL184 feeding increases hepatic β‐oxidation, also leading to diminished inflammation in the intestine. (A) Gene expression of *Pparγ1*, *Pparγ2*, and *Cd36* and (B) *Pparα*, *Cpt1α*, *Pgc1α* (in trend), *Hmox1*, and *Nrf2* (in trend) is up‐regulated. (C) Hepatic ATP content increased in mitochondria but not in cytosol. (D) Intestinal gene expression of *Pparα* and *Cpt1α* is up‐regulated, whereas *Pparγ2* and *Fgf15* remained unchanged; inflammatory markers *F4/80* and *Cox2* are strongly decreased. (E) Gas chromatography quantification of polyunsaturated FAs in the intestine from *Mdr2 ^−/−^* mice fed JZL184 evidenced enrichment of AA. (F) Microbiome analysis showed changed microbial composition in *Mdr2* ^−/−^ mice fed JZL184, with reduced abundance of Ruminococcaceae compared to *Mdr2* WT. Results are expressed as mean ± SD. *P < 0.05 for *Mdr2^−/−^* versus *Mdr2^−/−^* JZL184‐fed mice (n = 9). Abbreviation: NMDS, nonmetric multidimensional scaling.

We next aimed to verify if MGL pharmacological inhibition ameliorates spontaneous cholestasis in *Mdr2^−/−^*. Mice received 4 weeks of either a chow diet or JZL184 treatment. Consistent with our previous finding in DDC‐fed MGL^−/−^ mice, JZL184 was protective in *Mdr2^−/−^* as evidenced by diminished serum levels of ALT (trend also for AST), AP, BA (Fig. 5A,B), nonesterified FAs (Supporting Fig. S3A), and H&E (Fig. 5C). Fibrosis was also diminished as shown by sirius red staining (Fig. 5C) and by quantification of hepatic hydroxyproline content (Fig. 5D). Gene expression analysis evidenced diminished *Col1α1*, *Ck19* (also IHC; Fig. 5F), *Vcam‐1*, and *Cyp7a1* expression (Fig. 5E) and OPN staining (Supporting Fig. S2C). Inflammation was also attenuated by MGL inhibition in *Mdr2^−/−^*, with decreased expression of proinflammatory markers such as *Mcp1* (Fig. 5E) and amounts of Mac‐2^+^ (Fig. 5F) and CD11b^+^ (Supporting Fig. S2D) cells in IHC staining. FAs and cholesterol synthesis remained unaffected as reflected by *Srepb1c* and *Srebp2* expression (not shown). Notably, *Pparγ1 was* down‐regulated, whereas *Pparγ2*, *Cd36*, *Pparα*, *Pgc1α*, and *Cpt1α* gene expression was increased in *Mdr2^−/−^* mice fed JZL184 (Fig. 6A,B). *Hmox1* and *Nrf2* were up‐regulated (Fig. 6B), as was hepatic ATP content, in the mitochondria of *Mdr2^−/−^* mice fed JZL184 (Fig. 6C). Intestinal gene expression analysis showed increased *Pparα*, *Cpt1α*, and *Pparγ2* with unchanged *Fgf15* and diminished inflammation as reflected by *F4/80* and *Cox2* expression (Fig. 6D) and Mac‐2 staining (Supporting Fig. S2E). In keeping with our findings in MGL^−/−^ mice, AA accumulated in the intestine of *Mdr2^−/−^* mice fed JZL184 (Fig. 6E), pointing toward a potential role of the latter in intestinal inflammation. Furthermore, JZL184 induced changes in the microbiome composition in *Mdr2^−/−^* (Fig. 6F), showing a reduced abundance of Ruminococcaceae compared to WT. Hepatobiliary bile flow, biliary HCO_3_^–^ output, and biliary BA output remained unremarkable in mice fed JZL184 (Supporting Fig. S3B,C). Importantly, protein expression of BA transporters showed increased uptake (Ntcp) and canalicular export capacity (Bsep/Mrp2) and unchanged basolateral export (Mrp3/4) (Supporting Fig. S3D,E) in *Mdr2^−/−^* mice fed JZL184 (in line with the decreased plasma BA). Altogether, our data confirm the protective effect of MGL inhibition in a second model of cholestasis liver injury.

### MGL Inhibitors Mitigates Cholestatic Injury After DDC Challenge

**Figure 7:**
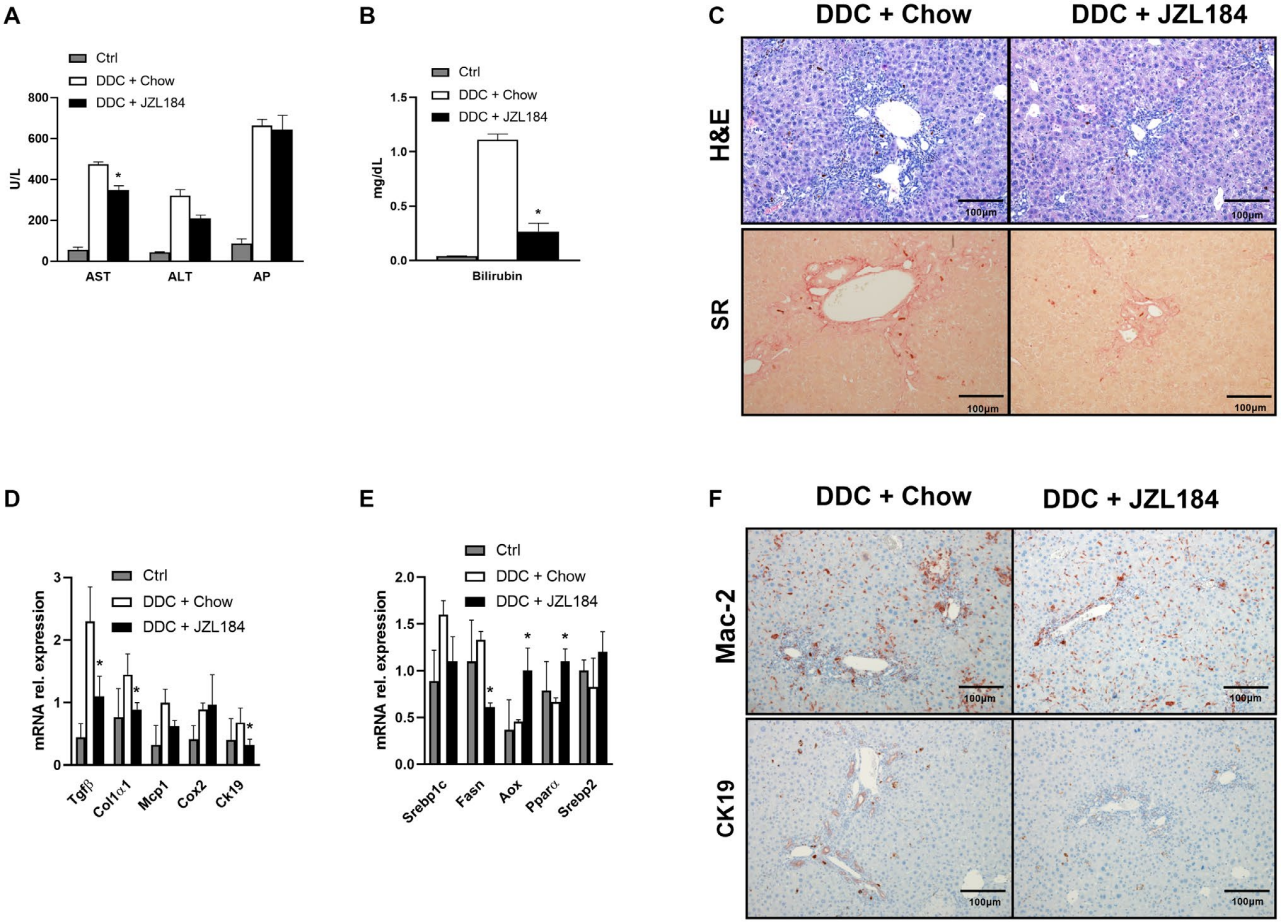
The MGL inhibitor JZL184 mitigates cholestatic injury after DDC feeding. WT mice received 2 weeks of DDC feeding and 4 days of JZL184 to investigate disease resolution. (A) Serum biochemistry reflects diminished levels of serum transaminases (ALT and trend for AST but unchanged AP) and (B) bilirubin. (C) Representative H&E images (×10 magnification) with improved liver histology and ameliorated fibrosis (sirius red) in WT animals fed DDC+JZL184. (D) Hepatic gene expression of profibrogenic markers *Tgfβ* and *Col1α1* diminished, while proinflammatory cytokines/chemokines *Mcp1* and *Cox2* remained unchanged and *Ck19* diminished. (E) Hepatic gene expression of *Srebp1c* and *Srebp2* was unchanged, *Fasn* diminished, whereas *Aox* and *Pparα* increased. (F) Representative images of Mac‐2 and CK19 staining in liver tissue showing ameliorated inflammation and cholangiocyte reactive phenotype. Gray bars represent control mice fed chow; white bars represent DDC + chow diet; black bars represent mice fed the DDC + JZL184 diet. Results are expressed as mean ± SD. *P < 0.05 for WT DDC + chow versus DDC + JZL184. Abbreviations: Ctrl, control; SR, sirius red.

In order to evaluate whether pharmacological inhibition of MGL accelerates regression of cholestatic liver injury after DDC feeding, we fed WT mice the DDC diet for 2 weeks and analyzed disease regression after 4 days of either chow or JZL184 treatment. Liver transaminases showed a significant decrease in AST and a trend for ALT with unchanged AP (Fig. 7A), while plasma bilirubin was significantly reduced in the JZL184‐fed group versus the group fed chow (Fig. 7B); TG and cholesterol levels remained unchanged (not shown). IHC staining showed diminished ductular reaction for H&E and decreased fibrosis on sirius red staining (Fig. 7C). Expression analysis of proinflammatory and profibrogenic genes showed decreased *Col1a1*, *Tgfb*, *Mcp1*, and *Ck19* and unchanged *Cox2* (Fig. 7D). Lipid metabolic genes decreased for *Srebp1c* and FA synthase (*Fasn*) and increased for *Aox* and *Pparα*, whereas *Srebp2* remained unchanged (Fig. 7E). Notably, Mac‐2 and CK19 staining intensity was reduced in mice receiving JZL184 after the DDC diet (Fig. 7F). Altogether, these results suggest that MGL inhibition could mitigate liver injury after DDC challenge.

### Silencing MGL Up‐Regulates PPAR‐α and PPAR‐γ Resulting in Diminished Intestinal Inflammation

**Figure 8:**
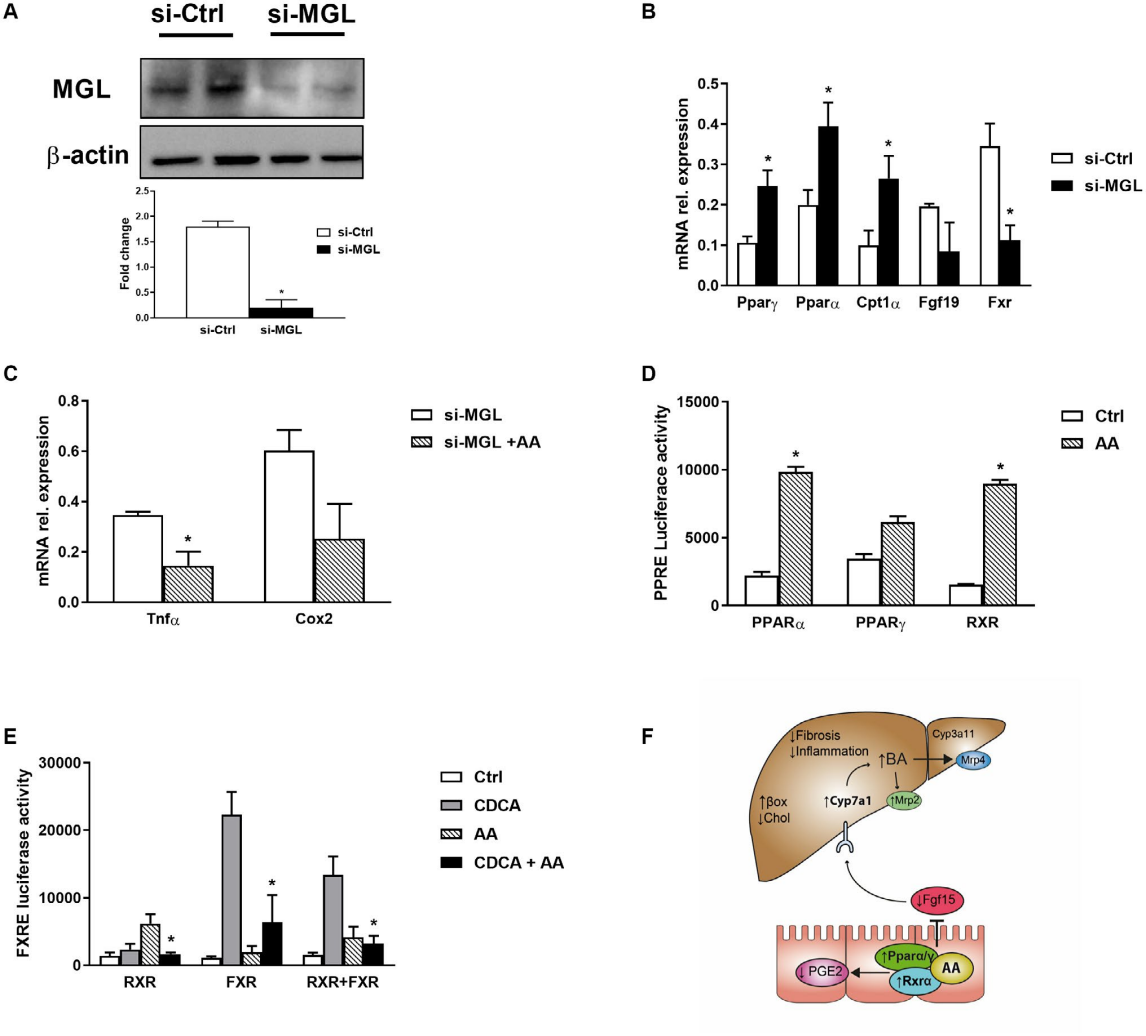
Silencing MGL in intestinal cells up‐regulates PPAR‐α and PPAR‐γ *in vitro*, and AA binds PPARs/RXR, diminishing inflammation. MGL was silenced in Caco2 cells. (A) Representative western blot with corresponding Image J quantification showing successful silencing at 5 µM concentration. (B) Caco2 siMGL had induction of *Pparγ*, *Pparα*, and *Cpt1α* with unchanged *Fgf19* gene expression and decreased *Fxr.* (C) Gene expression of proinflammatory genes *Tnfα* and *Cox2* down‐regulated after treatment with AA in siMGL Caco2 cells. (D) Transfection of PPARα, PPARγ, and RXR showed increased luciferase activity of PPRE after AA incubation. (E) Cotransfection of RXR/FXR shows that AA antagonizes induction of FXRE by CDCA. Results are expressed as mean ± SD of three independent experiments in duplicate, *P < 0.05 for siMGL versus siMGL+CDCA and AA treatment versus control or CDCA+AA treatment versus CDCA alone. (F) MGL deletion ameliorates cholestatic liver disease induced by DDC challenge diminishing fibrosis, inflammation, and FA metabolism/oxidation in the liver. It also induces BA synthesis and detoxification as shown by Cyp7a1/Cyp3a11 induction and BA transport (Mrp4) with unchanged Bsep and increased Mrp2. In the intestine, accumulation of AA binds NRs PPAR‐α, PPAR‐γ, and RXRα, resulting in anti‐inflammatory effects as further demonstrated by diminished PGE_2_ content. Abbreviations: Chol, cholesterol; Ctrl, control; βox, β‐oxidase.

To further explore the molecular mechanism, we silenced MGL with siRNA in human Caco‐2 cells (Fig. 8A). When MGL was silenced, gene expression of the NRs *Pparγ*, *Pparα*, and target gene *Cpt1α* was up‐regulated, while *Fxr* was down‐regulated with a similar trend for *Fgf19* (Fig. 8B). FXR agonism by CDCA was blunted after siMGL in Caco‐2 as demonstrated by reduced *Fxr* and downstream targets such as *Fgf19*, small heterodimer protein, ileal BA binding protein, and gel shift assay (Supporting Fig. S4A,B). For IHHs, *Fgf19* and *Fxr* were diminished, for cholangiocytes (BECs) only *Fxr* was diminished, while no changes were seen for hepatic stellate cells (LX2) and macrophages (U937) (Supporting Fig. S4A). Caco‐2 cells treated with AA showed diminished inflammation as evidenced by *Tnfα* and *Cox2* (trend) gene expression (Fig. 7C). Retinoid X receptor alpha (RXRα; NR2B1), a member of the NR superfamily, is a promiscuous heterodimeric partner for many NRs, including PPARs and FXR.^22^ A transfection assay with PPAR‐α, PPAR‐γ, and RXR on their response element demonstrated up‐regulation of their respective luciferase activity after treatment with AA (Fig. 8D), accumulating in the intestine after MGL deletion/inhibition (Figs. 4B and 6E). In other liver cell types peroxisome proliferator–response element (PPRE) luciferase activity was up‐regulated after AA treatment for PPAR‐α only in IHHs and PPAR‐γ in LX2 cells, whereas BECs and U937 cells were unresponsive (Supporting Fig. S4C). In addition, the FXR/RXR heterodimer binds to FXR response elements (FXREs), which in turn control the response to BAs. RXRα is also able to bind to FAs such as docosahexaenoic acid and AA.^23^ Because RXR is the heterodimeric partner of FXR, we tested whether AA would disrupt FXR signaling in the intestine. Indeed, we demonstrate that RXR agonism induced by AA in turn antagonizes ligand‐bound FXR‐induced expression of FXRE luciferase activity (Fig. 8E). Altogether, these data demonstrate that MGL deletion favors the accumulation of AA in the intestine, triggering protective mechanisms through NR activation counteracting cholestatic liver disease progression, as summarized in Fig. 8F.

## Discussion

Cholestatic liver diseases are characterized by impaired bile formation, resulting in hepatic accumulation of BA, cholesterol, and bilirubin^24^; once BAs accumulate in high concentrations, they can leak from canaliculi and bile ducts, thus causing hepatic cell death, inflammation, fibrosis, and cancer.^25^ Cholestatic liver diseases are also known to impair the intestinal absorption of FAs and sterols due to poor micellar formation secondary to reduced bile formation and excretion.^26^ An important feature of PSC is its association with inflammatory bowel disease and ulcerative colitis.^27^ In fact, a leaky gut allows bacterial products to reach the liver and cause inflammation, making the gut–liver axis a pivotal player in disease pathogenesis.^27^ We hypothesized that MGL invalidation may represent an attractive target by modulating FA and BA metabolism as well as providing ligands for NRs in the liver and intestine, ultimately driving protective mechanisms to counteract biliary injury and fibrosis. While previous reports evidenced the pivotal role of MGL in tumor growth, in metabolism,^28^ in oncogenic signaling such as invasion and migration,^28^ and as a prognostic indicator for hepatocellular carcinoma,^29^ others have shown that MGL deletion resulted into colorectal cancer growth inhibition,^30^ also attenuating acute lung injury in mice^31^ and improving adipose tissue inflammation and insulin resistance in obese mice.^8^ In addition, we recently showed that MGL invalidation strongly decreases fibrogenesis due to autophagy‐mediated anti‐inflammatory properties.^12^ However, very little is known about the role of MGL in cholestatic diseases including PSC, a cholangiopathy with the largest unmet medical need due to lack of effective pharmacological therapies.^3^ To address this question, we challenged MGL^−/−^ mice with acute DDC feeding for 2 weeks, discovering that MGL deletion decreases ductular reaction with diminished reactive cholangiocyte phenotype, fibrosis, and inflammation. FA synthesis and uptake were up‐regulated, coupled with increased mitochondrial β‐oxidation, suggesting a futile circle of hydrolysis and esterification in the liver. BA transport was increased, thus explaining the relatively increased amount of plasma BAs in MGL^−/−^ mice fed DDC, although *Mdr2^−/−^* mice showed decreased BA. This suggests that bile duct injury may be improved without affecting cholestasis/BA homeostasis. Importantly, ileal inflammation was diminished due to increased PPAR‐α and PPAR‐γ in DDC‐treated MGL^−/−^ mice; a more detailed analysis of molecular FA species revealed enrichment of the AA intestinal pool in MGL^−/−^ mice due to increased expression of alternative lipid‐hydrolyzing enzymes Abhd6 and Abhd12. In addition, we explored the relevance of our findings in a therapeutic setting, treating *Mdr2^−/−^* mice (an established model of PSC developing spontaneous sclerosing cholangitis and biliary fibrosis), with the MGL inhibitor JZL184. Importantly, MGL inhibition confirmed the protective effects in *Mdr2^−/−^* mice, which showed reduced biliary fibrosis and inflammation. FAs and cholesterol synthesis remained unaffected, while FAs and β‐oxidation improved in JZL184‐treated mice. Intriguingly, gas chromatographic analysis of FA species revealed AA accumulation in the intestine with diminished inflammation and NR up‐regulation after 4 weeks of JZL184 feeding. Gut microbiome analysis evidenced reduced abundance of Proteobacteria in MGL^−/−^ mice fed DDC, which are known to be overexpressed in inflammatory conditions; and children with nonalcoholic steatohepatitis have shown gradual increase in Proteobacteria in comparison to healthy controls.^32^, ^33^ In *Mdr2^−/−^* mice, Ruminococcaceae—positively correlated with the production of beneficial short‐chain FAs and AA,^34^ which show anti‐inflammatory properties in the intestine—were restored by JZL184 feeding to levels comparable to WT mice.

In WT mice, cholestatic liver injury was mitigated by JZL184 treatment after 2 weeks of DDC feeding. Intriguingly, the different plasma BA response to injury in the MGL^−/−^ DDC and *Mdr2^−/−^* models could be linked to the etiopathogenesis of cholestatic liver injury because *Mdr2^−/−^* mice are known to develop cholestasis from toxic biliary BA composition, while DDC represents a more direct xenobiotic induced form of bile duct injury. Mechanistically, *in vitro* experiments provided compelling evidence that MGL silencing leads to PPAR‐α and PPAR‐γ up‐regulation, while AA binds PPAR‐α, PPAR‐γ, and RXR in luciferase assay, also inducing antagonism of FXR activity. This is in line with a previous report demonstrating that RXR agonism induces respective antagonism of FXR activity due to absence of coactivator recruitment and decreased DNA binding.^35^ NRs are known actors in PSC and PBC; PPARα is, for instance, involved in BA homeostasis, repressing its synthesis through hepatocyte nuclear factor 4 alpha, which binds to the Cyp7a1 promoter.^36^ PPARα ligands such as fibrates repress BA synthesis and promote phospholipid secretion into bile through induction of Mdr3.^37^ PPARγ instead is an important target for inflammatory cholestasis because of its crucial role in attenuating inflammation. In a lipopolysaccharide (LPS) model of inflammatory cholestasis, treatment with glitazones was shown to attenuate the repression of Ntcp, Bsep, and Cyp3a11.^38^ Finally, RXR was shown to modulate inflammation in acute colitis,^39^ whereas FXR activation was demonstrated to limit hepatic inflammation by inhibiting the nuclear factor kappa Β–mediated inflammatory response,^40^ also restricting fibrosis.^41^ Although current strategies in cholestasis favor FXR agonists,^42^ FXR antagonism was also found to be beneficial in obesity and insulin resistance through gut–liver axis involvement and microbiome modulation.^43^ Moreover, mice lacking FXR are protected from cholestatic injury due to adaptive induction of alternative (FXR‐independent) detoxification pathways regulated by pregnane X receptor and constitutive androstane receptor.^44^, ^45^ A key relevant finding of this study is that MGL products ameliorate intestinal inflammation through NR signaling and AA binding, and this might be of great relevance in the context of intestinal disorders connected to cholestatic diseases. COX‐2 oxidizes 2‐AG to generate prostaglandin glycerol esters (PG‐G) through pathways closely matching those of the respective prostaglandins formed from AA.^46^ Importantly, it has been demonstrated that, under specific conditions, 2‐AG‐derived PG‐G species are formed by COX‐2 action and that these metabolites likely have bioactivities of their own, mediated through as yet unknown nonprostaglandin receptors.^47^ For instance, pharmacologic inhibition of Abhd6 in an LPS murine model induced 2‐AG accumulation in macrophages and channeling to COX‐2‐derived anti‐inflammatory PG‐G species, including PGD_2_‐G, a precursor of 15d‐PGJ_2_‐G.^48^ Moreover, *in vivo* administration of PGD_2_‐G was shown to reduce LPS‐induced inflammation.^48^ Another study revealed that Abhd6 hydrolyzes PGD_2_‐G, whereas MGL prefers 15d‐PGJ_2_‐G, which in turn activates the Nrf2 signaling pathway, in line with our data in the liver.^49^ In light of our findings, AA accumulation might not only channel PGD_2_‐G generation but could also concomitantly result in an anti‐inflammatory effect due to direct binding to NRs. This should be a viable hypothesis to be tested in future *in vivo* studies.

In summary, we demonstrate that MGL deletion has crucial roles in the regulation of lipid and BA homeostasis, rescuing mitochondrial respiration/antioxidant response and ameliorating cholestatic disease. Intestinal inflammation was positively affected by lipid signaling, microbial changes, and AA accumulation in both animal models, pointing to a shared molecular mechanism which involved RXR binding, activation of PPARs, and FXR competition.^50^ Therefore, our findings may open therapeutic horizons in treating cholestatic liver diseases such as PSC through the gut–liver axis, suggesting the potential for selective MGL antagonists as a future treatment strategy.

## Author Contributions

M.T. was responsible for study design, acquisition of data, analysis and interpretation of data, and manuscript writing. F.V.B., C.D.F., M.B., O.A.H.O.R., H.J.V., N.A., V.K., T.S., H.S., and A.H. were responsible for acquisition and analysis of data. T.C. was responsible for analysis and interpretation of data and manuscript writing. R.Z. was responsible for analysis and interpretation of data. S.L. and M.T. were responsible for study concept, analysis and interpretation of data, manuscript writing, and obtaining funding.
