# Supplementary material for: Monoacylglycerol Lipase Inhibition Protects From Liver Injury in Mouse Models of Sclerosing Cholangitis
Source: Hepatology. 2019 Dec 30;71(5):1750–65. doi: 10.1002/hep.30929 (PMC7317927; doi:10.1002/hep.30929)

Monoacylglycerol lipase inhibition protects from liver injury in mouse models of sclerosing cholangitis

Matteo Tardelli [1], Francesca V. Bruschi [1], Claudia D. Fuchs [1], Thierry Claudel [1], Nicole Auer [1], Victoria Kunczer [1], Maximilian Baumgartner [2], Onne A.H.O. Ronda [3], Henk Jan Verkade [3], Tatjana Stojakovic [4], Hubert Scharnagl [5], Aida Habib [6,7], Robert Zimmermann[8], Sophie Lotersztajn [6] and Michael Trauner [1]

[1] Hans Popper Laboratory of Molecular Hepatology, Division of Gastroenterology and Hepatology, Department of Internal Medicine III, Medical University of Vienna, Austria.

[2] Division of Gastroenterology and Hepatology, Department of Internal Medicine III, Medical University of Vienna, Austria.

[3] Center for Liver, Digestive and Metabolic Diseases, Departments of Pediatrics, University Medical Center Groningen, University of Groningen, Groningen, The Netherlands.

[4]Clinical Institute of Medical and Chemical Laboratory Diagnostics, University Hospital Graz, Austria.

[5]Clinical Institute of Medical and Chemical Laboratory Diagnostics, Medical University of Graz, Austria.

[6] Université de Paris, Centre de Recherche sur l'Inflammation (CRI), INSERM,UMR1149, CNRS, ERL 8252, F-75018 Paris, France.

[7] Department of Biochemistry and Molecular Genetics, American University of Beirut, Lebanon.

[8] Institute of Molecular Biosciences, University of Graz, Austria.

Table of contents:

- Supplementary material and methods;
- Supplementary figure legends;
- References.

Supplementary material and methods:

Hepatic hydroxyproline content

To quantify liver fibrosis, hepatic hydroxyproline was measured from a standardized liver lobe as described previously (1).

ELISA measurement of PGE_2

The concentrations of PGE_2 in the intestine, liver and plasma were determined by quantitative ELISA as previously described (2,3) according to the manufacturer's directions - Enzo Biosciences (Lausen, Switzerland).

Hepatic ATP content

Hepatic ATP content was measured by colorimetric assay in liver homogenates according to the manufacturer's instructions, ATP assay kit (Abcam, Cambridge, UK). Mitochondria fraction and cytosol were separated with multi-step centrifugation at high speed according to manufacturer´s protocol (mitochondria isolation kit for tissue, Mitosciences, Oregon, USA).

RNA extraction and quantitative reverse transcriptase polymerase chain reaction (qRT-PCR) analysis

Tissue and cells were homogenized in TRIzol reagent (Thermo Fisher Scientific) and RNA isolated according to the manufacturer´s protocol. Total RNA (1 µg) was retro-transcribed into cDNA using Superscript II and random hexamer primers (Thermo Fisher Scientific). Gene expression was analyzed by quantitative real-time PCR on an ABI Step One Plus cycler using assays-on-demand kits (TaqMan® Gene Expression Assay, Thermo Fisher Scientific). Each reaction was performed in duplicate and the value of the gene of interest was normalized to human ubiquitin C expression. The comparative threshold cycle (CT) method was used to calculate the relative expression.

Bile flow measurement and HCO_3^-/BA output

Before harvesting, mice were anesthetized with a mixture of Ketamin and Xylazol intraperitoneally. The common bile duct was ligated, and the gallbladder cannulated for bile collection. After an equilibration period, bile was collected for the following 20 minutes in pre-weighed test tubes. Bile flow was determined gravimetrically and normalized to liver weight (4,5). For additional 40 minutes bile was collected in test tubes under mineral oil for determination of HCO_3^-, total carbon dioxide and pH using an automated blood gas analyzer. Biliary bile acid concentration was measured enzymatically using a colorimetric bile acid kit according to manufacturer´s protocol (DiaSys Diagnostic Systems GmbH, Holzheim Germany).

Supplementary figure legends:

Suppl. Figure 1. MGL$^{-/-}$ fed DDC are less inflamed than WT. (A) Plasma bile acids level for MGL$^{-/-}$ fed DDC. (B) Hepatic gene expression of *F4/80, Opn* and *Vcam-1*

downregulated highlight reduced inflammation. (C) Image J quantification of CD11b IF for all groups and (D) OPN staining. (E) Hepatic gene expression of *Ppar*δ, *Lipin2* were upregulated; (F) intestinal gene expression of *Asbt, Ost*α*, Ostβ* remained unchanged in MGL$^{-/-}$ fed DDC. Results are expressed as mean ± S.D.; * p<0.05 for MGL$^{-/-}$ DDC versus WT DDC (n=8).

Suppl. Figure 2. MGL inhibition diminishes intestinal and hepatic inflammation. (A) Representative images (10x magnification) for intestinal H&E and Mac-2 in DDC fed WT and MGL$^{-/-}$. (B) PGE2 content in plasma was diminished in MGL$^{-/-}$ fed DDC vs WT DDC. (C) OPN staining in *Mdr2*$^{-/-}$ mice and (D) Image J quantification of CD11b IF. (E) Representative images for intestinal H&E and Mac-2 in *Mdr2*$^{-/-}$ and *Mdr2*$^{-/-}$ JZL184 fed mice. * p<0.05 for MGL$^{-/-}$ DDC versus WT DDC (n=8) or *Mdr2*$^{-/-}$ versus *Mdr2*$^{-/-}$ JZL184 fed mice (n=9).

Suppl. Figure 3. Mdr2$^{-/-}$ fed JZL184 show unchanged biliary BA/HCO_3^- output but increased BA transport. (A) Serum NEFA levels in *Mdr2*$^{-/-}$ versus *Mdr2*$^{-/-}$ JZL184 fed mice. (B) Bile flow, (C) biliary HCO_3^- output and biliary BA output remain unchanged under JZL184 (n=4). (D) Representative western blot with corresponding densitometry (E) of BA transporters showing increased uptake systems (Ntcp, although unchanged Oatp1) and augmented export (Bsep and Mrp2/3) (n=3). Calnexin was used as loading control. * p<0.05 for *Mdr2*$^{-/-}$ versus *Mdr2*$^{-/-}$ JZL184 fed mice.

Suppl. Figure 4. AA binds to NRs in IHH/LX2 and FXR agonism is inhibited by si-MGL. (A) Caco-2 si-MGL had suppression of *Fgf19, Shp, Ibabp* and *Fxr* gene expression. In IHH si-MGL *Fxr* and *Fgf19* were also diminished after CDCA treatment. For BECS *Fxr* diminished whereas si-MGL had no effect in LX2 and U937 after CDCA agonism. (B) Gel Shift assay showing: 1 cytoplasm control, 2 nucleus control, 3 cytoplasm si-MGL, 4 Nucleus si-MGL, 5 cytoplasm CDCA, 6 nucleus CDCA, 7

cytoplasm si-MGL + CDCA, 8 nucleus si-MGL + CDCA, 9 nucleus si-MGL + CDCA +FXR antibody. (C) Transfection of PPARα, PPARγ and RXR showed increase luciferase activity of PPRE for PPARα in IHH and PPARγ in LX2. * $p<0.05$ for si-MGL vs si-MGL+CDCA and AA treatment vs control.

References:

1. Fickert P, Wagner M, Marschall HU, Fuchsbichler A, Zollner G, Tsybrovskyy O, et al. 24-norUrsodeoxycholic acid is superior to ursodeoxycholic acid in the treatment of sclerosing cholangitis in Mdr2 (Abcb4) knockout mice. Gastroenterology. 2006;130:465–481.
2. Yao M, Kargman S, Lam EC, Kelly CR, Zheng Y, Luk P, et al. Inhibition of cyclooxygenase-2 by rofecoxib attenuates the growth and metastatic potential of colorectal carcinoma in mice. Cancer Res. 2003;
3. Wang X, Shaw DK, Hammond HL, Sutterwala FS, Rayamajhi M, Shirey KA, et al. The Prostaglandin E2-EP3 Receptor Axis Regulates Anaplasma phagocytophilum-Mediated NLRC4 Inflammasome Activation. PLoS Pathog. 2016;
4. Fuchs CD, Paumgartner G, Mlitz V, Kunczer V, Halilbasic E, Leditznig N, et al. Colesevelam attenuates cholestatic liver and bile duct injury in *Mdr2* $^{-/-}$ mice by modulating composition, signalling and excretion of faecal bile acids. Gut [Internet]. 2018;gutjnl-2017-314553. Available from: http://gut.bmj.com/lookup/doi/10.1136/gutjnl-2017-314553
5. Fuchs CD, Paumgartner G, Wahlstraem A, Schwabl P, Reiberger T, Leditznig N, et al. Metabolic preconditioning protects BSEP/ABCB11-/- mice against cholestatic liver injury. J. Hepatol. 2017;

Suppl. Figure 1. Tardelli et al.

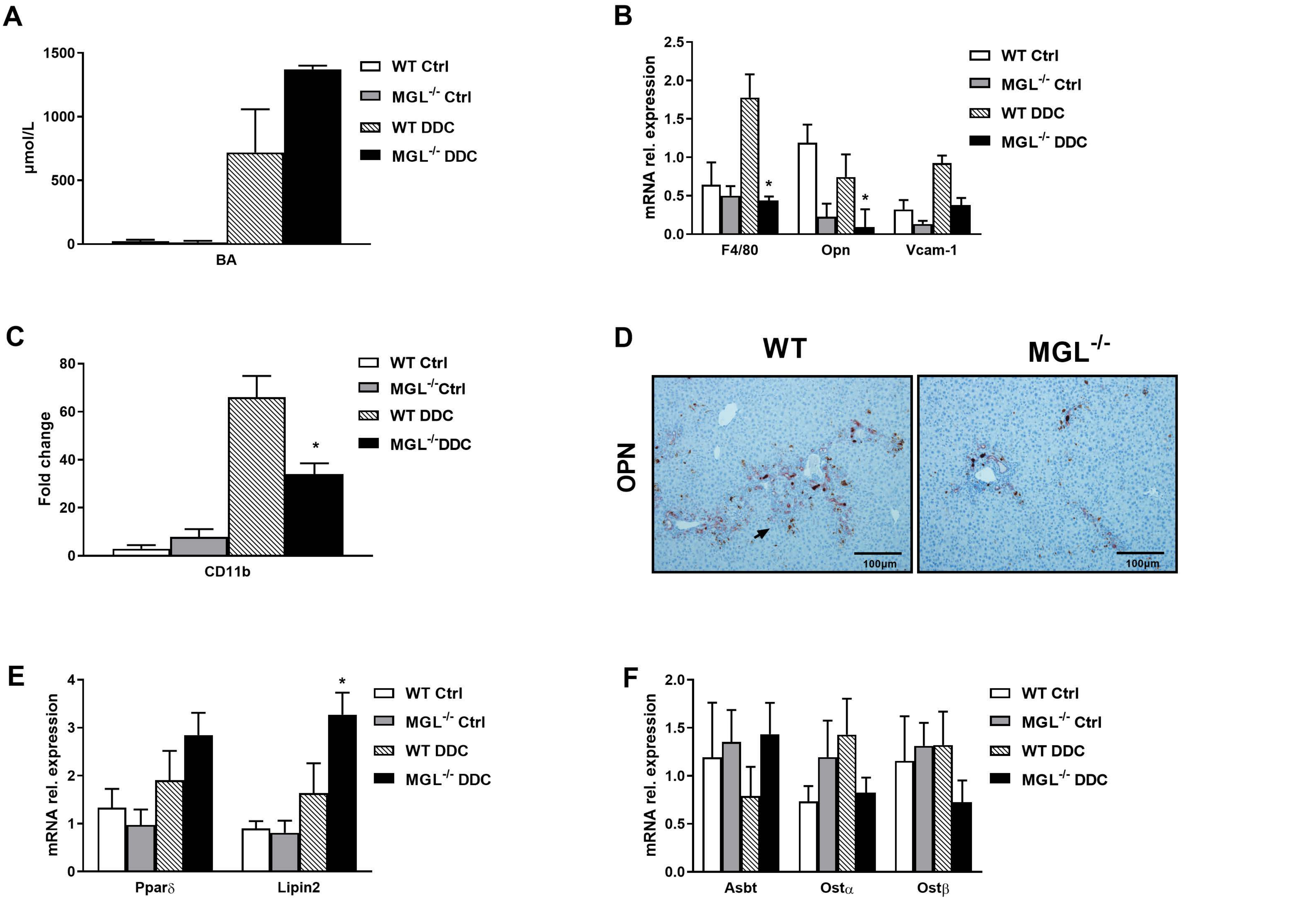

Suppl. Figure 2. Tardelli et al.

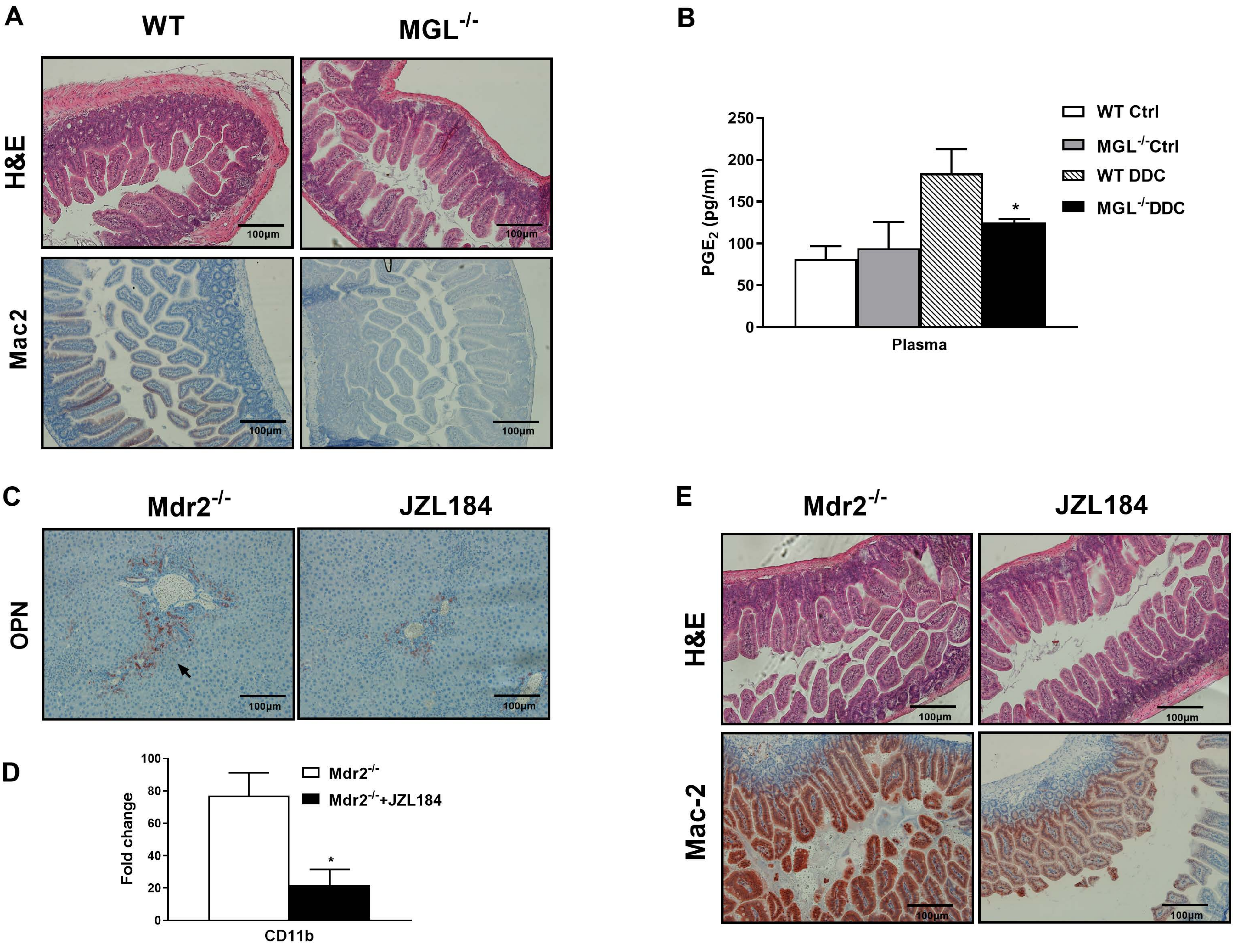

Suppl. Figure 3. Tardelli et al.

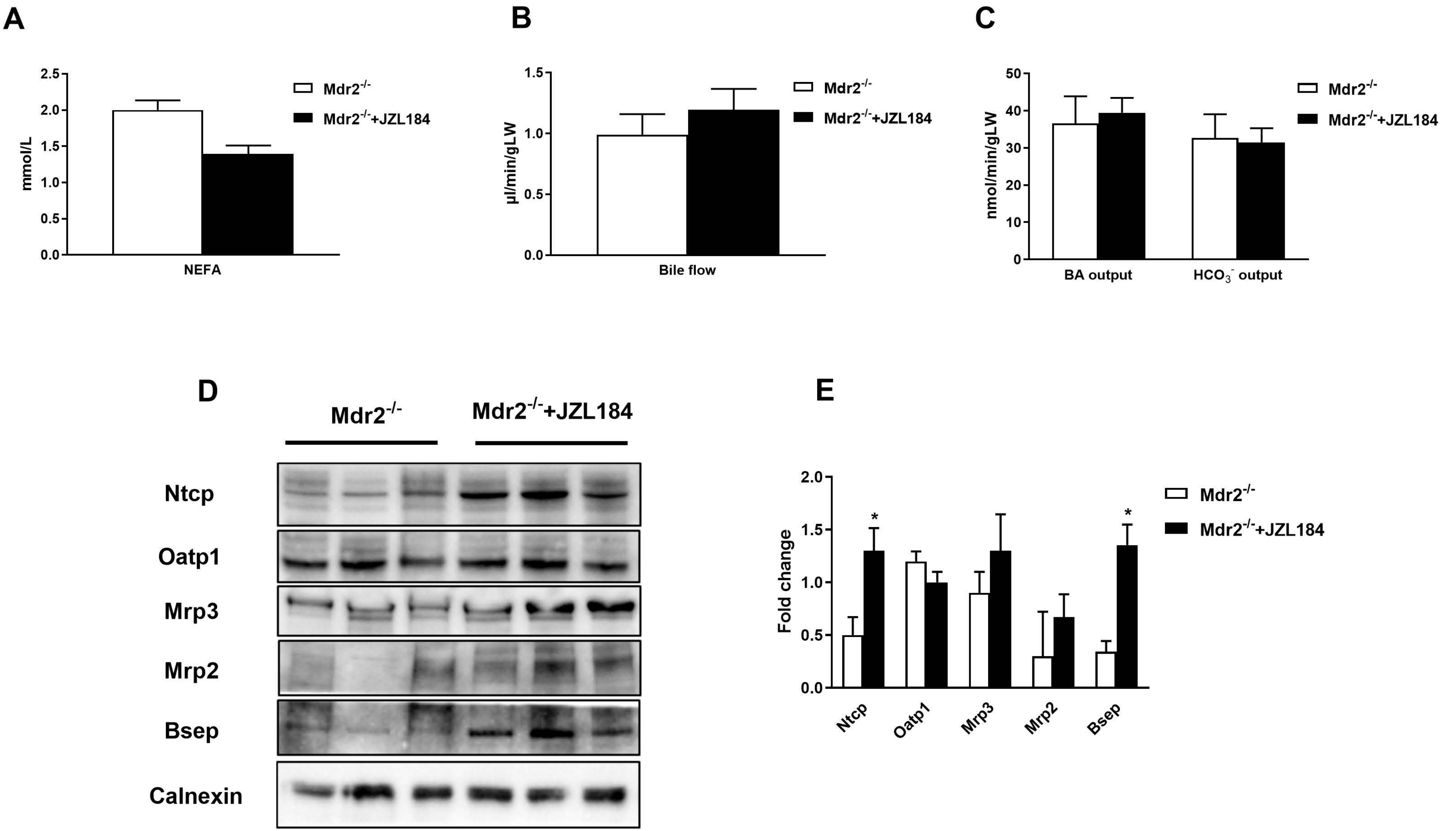

Suppl. Figure 4. Tardelli et al.

A

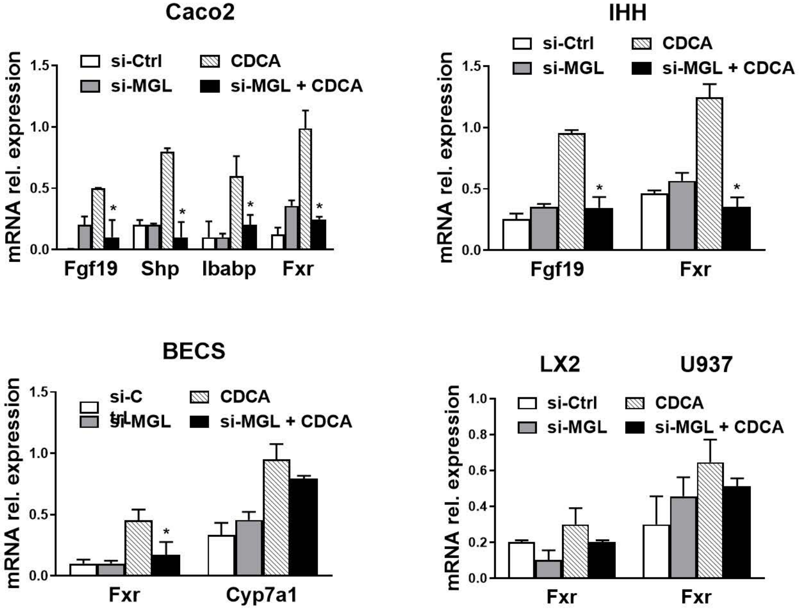

B

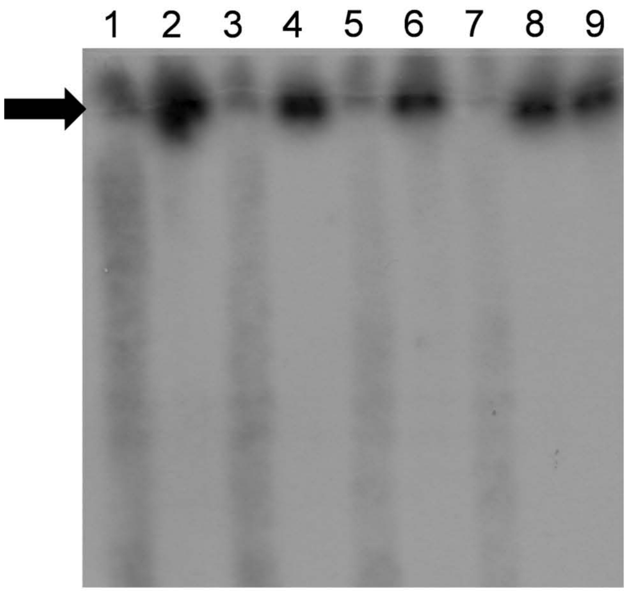

C

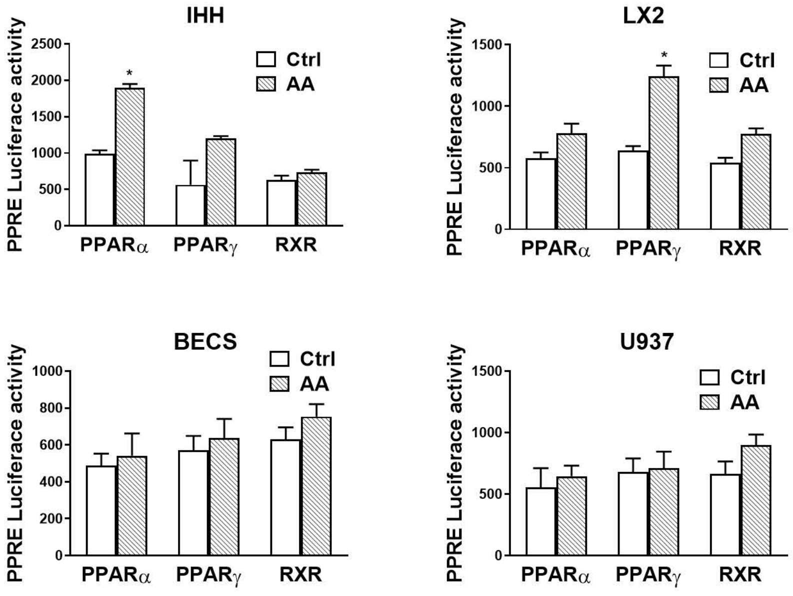

Supplement: Supplementary file 1 [file HEP-71-1750-s001.pdf]
